# Antioxidants, Gut Microbiota, and Cardiovascular Programming: Unraveling a Triad of Early-Life Interactions

**DOI:** 10.3390/antiox14091049

**Published:** 2025-08-26

**Authors:** Chien-Ning Hsu, Ying-Jui Lin, Chih-Yao Hou, Yu-Wei Chen, Guo-Ping Chang-Chien, Shu-Fen Lin, You-Lin Tain

**Affiliations:** 1 Department of Pharmacy, Kaohsiung Chang Gung Memorial Hospital, Kaohsiung 833, Taiwan; cnhsu@cgmh.org.tw; 2 Department of Pharmacy, Kaohsiung Municipal Ta-Tung Hospital, Kaohsiung 801, Taiwan; 3 School of Pharmacy, Kaohsiung Medical University, Kaohsiung 807, Taiwan; 4 Division of Critical Care, Department of Pediatrics, Kaohsiung Chang Gung Memorial Hospital and Chang Gung University College of Medicine, Kaohsiung 833, Taiwan; rayray@adm.cgmh.org.tw; 5 Division of Cardiology, Department of Pediatrics, Kaohsiung Chang Gung Memorial Hospital and Chang Gung University College of Medicine, Kaohsiung 833, Taiwan; 6 Department of Respiratory Therapy, Kaohsiung Chang Gung Memorial Hospital and Chang Gung University College of Medicine, Kaohsiung 833, Taiwan; 7 Department of Early Childhood Care and Education, Cheng Shiu University, Kaohsiung 833, Taiwan; 8 Department of Seafood Science, National Kaohsiung University of Science and Technology, Kaohsiung 811, Taiwan; chihyaohou@nkust.edu.tw; 9 Department of Food Science and Biotechnology, National Chung Hsing University, Taichung 402, Taiwan; d112043001@mail.nchu.edu.tw; 10 Department of Pediatrics, Kaohsiung Chang Gung Memorial Hospital, Kaohsiung 833, Taiwan; 11 Center for Environmental Toxin and Emerging-Contaminant Research, Cheng Shiu University, Kaohsiung 833, Taiwan; guoping@csu.edu.tw (G.-P.C.-C.); linsufan2003@gmail.com (S.-F.L.); 12 Super Micro Mass Research and Technology Center, Cheng Shiu University, Kaohsiung 833, Taiwan; 13 Institute of Environmental Toxin and Emerging-Contaminant, Cheng Shiu University, Kaohsiung 833, Taiwan; 14 Department of Pediatrics, Kaohsiung Municipal Ta-Tung Hospital, Kaohsiung 801, Taiwan; 15 College of Medicine, Chang Gung University, Taoyuan 333, Taiwan

**Keywords:** oxidative stress, antioxidants, cardiovascular disease, hypertension, gut microbiota, developmental origins of health and disease, short-chain fatty acid

## Abstract

Cardiovascular disease (CVD) remains the leading cause of global mortality, despite advances in adult-focused prevention and therapy. Mounting evidence supports the Developmental Origins of Health and Disease (DOHaD) paradigm, which identifies early-life exposures as critical determinants of long-term cardiovascular health. Among the key mechanistic pathways, oxidative stress and gut microbiota dysbiosis have emerged as central, interrelated contributors to cardiovascular programming. Prenatal and postnatal insults can induce sustained redox imbalance and disrupt microbial homeostasis. This disruption creates a feed-forward loop that predisposes offspring to CVD later in life. Antioxidants offer a promising reprogramming strategy by targeting both oxidative stress and gut microbiota composition. Preclinical studies demonstrate that maternal antioxidant interventions—such as vitamins, amino acids, melatonin, polyphenols, N-acetylcysteine, and synthetic agents—can restore redox homeostasis, modulate gut microbial communities, and attenuate cardiovascular risk in offspring. This review synthesizes current evidence on how oxidative stress and gut microbiota act together to shape cardiovascular trajectories. It also examines how antioxidant-based therapies may disrupt this pathological axis during critical developmental windows. Although human data remain limited due to ethical and practical constraints, advancing microbiota-targeted antioxidant interventions may offer a transformative approach to prevent CVD at its origins.

## 1. Introduction

Despite decades of progress in adult-focused management and therapeutic strategies [1,2], cardiovascular disease (CVD) continues to be the foremost cause of mortality globally [3]. Evidence is accumulating that events and exposures during fetal and early postnatal life can shape lifelong cardiovascular risk, a phenomenon described by the Developmental Origins of Health and Disease (DOHaD) hypothesis [4,5]. The DOHaD framework addresses the root causes of CVD by targeting critical early-life windows [6], offering a proactive, preventive, and intergenerational approach to reduce cardiovascular risk more effectively and equitably across populations. Understanding how prenatal and postnatal environments influence cardiovascular development—via factors such as maternal nutrition, stress, toxin exposure, disease, and early-life microbiota composition [7,8,9,10]—enables a shift from reactive adult care to a life-course strategy that prioritizes early prevention.

The fetal and early postnatal periods are especially vulnerable, representing stages of rapid growth and organ maturation during which adverse environmental exposures can lead to permanent alterations in cardiovascular structure and function—commonly referred to as cardiovascular programming [10,11]. This process involves a multifaceted interplay of mechanisms, including oxidative stress [12], hormonal imbalances [13], inflammation [14], epigenetic modifications [15], and gut microbiota dysbiosis [10]. These early-life disturbances reshape the developmental trajectory of the cardiovascular system, predisposing individuals to hypertension, vascular dysfunction, and heart disease in later life. Among these mechanisms, gut microbiota dysbiosis has emerged as a particularly central and modifiable contributor to CVD risk [11].

Evidence increasingly implicates the gut microbiota in CVD, primarily through its effects on metabolic balance, immune signaling, and inflammation [16,17,18]. Protective metabolites like short-chain fatty acids (SCFAs) enhance vascular integrity and regulate blood pressure (BP), while harmful products such as trimethylamine-N-oxide (TMAO) drive atherosclerosis and endothelial dysfunction [19,20]. Dysbiosis—an imbalance in microbial composition—can disrupt gut barrier integrity, leading to systemic inflammation and endothelial injury, both key processes in CVD pathogenesis. Furthermore, early-life disruptions in gut microbiota, as emphasized in the DOHaD framework, may program long-term cardiovascular risk [21,22]. Given its modifiable nature, the gut microbiota represents a promising target for early-life interventions and precision therapies aimed at reducing CVD risk across the lifespan [11].

Given that oxidative stress is a central pathogenic mechanism underlying CVD, antioxidants have been widely studied in both human and animal models as potential therapeutic agents [23]. While some interventions have shown promise in reducing oxidative biomarkers and improving endothelial function, large clinical trials have not consistently demonstrated significant reductions in major cardiovascular events [24]. The role of antioxidants in CVD and cardiovascular programming is related but distinct as they act at different stages of disease development—namely, adult treatment versus early-life prevention of future cardiovascular risk. Antioxidants have gained attention as a reprogramming strategy, which involves shifting the focus of intervention from disease management in adulthood to early-life prevention. This approach aims to halt or reverse adverse developmental programming processes, thereby reducing disease susceptibility later in life [25,26]. Emerging evidence from animal studies suggests that antioxidant supplementation during pregnancy and lactation may prevent cardiovascular dysfunction in offspring through developmental reprogramming, underscoring its potential as an early-life intervention within the DOHaD framework [27,28,29].

A bidirectional relationship exists between antioxidants and the gut microbiota: antioxidants can modulate microbial composition [30,31], while gut microbes enhance antioxidant bioavailability and activity [32,33]. Together, they synergistically regulate oxidative stress, inflammation, and metabolic pathways—key processes in CVD pathogenesis. Thus, targeting the antioxidant–microbiota axis may provide novel strategies for CVD prevention or mitigation, particularly through antioxidants rich diets and microbiota-directed therapies. This narrative review explores the role of antioxidants in the interplay between gut microbiota and cardiovascular programming, emphasizing their potential as a reprogramming approach to prevent CVD originating in early life.

## 2. Materials and Methods

This review aims to integrate current evidence on how antioxidants influence the gut microbiome and the potential consequences of these interactions for cardiovascular development and long-term disease risk, framed within the context of the DOHaD. The focus was on early-life mechanisms linking oxidative stress, gut dysbiosis, and CVD risk, with particular attention to antioxidant–microbiota interactions as potential reprogramming strategies. While oxidative stress and gut microbiota dysbiosis have each been implicated in CVD, their mechanistic interconnection—particularly in shaping long-term cardiovascular outcomes from early life—remains underexplored. By integrating evidence on how oxidative stress influences microbial composition and how microbiota-derived metabolites, in turn, modulate host redox balance, this review offers a novel perspective on a bidirectional oxidative stress–microbiota axis in CVD development. In addition, one of the objectives is to assess the translational potential of microbiota-targeted antioxidant interventions as future strategies for CVD prevention and therapy.

A comprehensive literature search was performed using PubMed, Scopus, and Web of Science databases to identify relevant peer-reviewed articles published up to June 2025. Search terms included combinations of the following keywords: “antioxidants”, “oxidative stress”, “gut microbiota”, “dysbiosis”, “cardiovascular disease”, “hypertension’, “developmental programming”, “DOHaD”, “reprogramming”, “early-life intervention”, “pregnancy”, “lactation”, “offspring”, “short-chain fatty acids”, “TMAO”, and “endothelial function”. Boolean operators (AND, OR) were applied to refine the search. Reference lists of key articles were manually screened to identify additional relevant studies.

Both preclinical (animal) and clinical (human) studies were included if they examined the role of antioxidants in modulating oxidative stress or cardiovascular outcomes, their effects on gut microbiota composition or function, or early-life exposures relevant to cardiovascular programming. Eligible publications consisted of original research articles, systematic reviews, and meta-analyses published in English. Studies were excluded if they were non-peer-reviewed (e.g., editorials or conference abstracts) or lacked relevance to early-life developmental programming or antioxidant–microbiota interactions. Given the interdisciplinary scope and heterogeneity of the available literature, a narrative review approach was chosen over a systematic or scoping format to facilitate a comprehensive and integrative exploration of emerging concepts and mechanisms spanning developmental biology, microbiome research, cardiovascular science, and nutrition. We used Napkin AI for generating the figures.

## 3. Distinct Roles of Gut Microbiota in Overt CVD and Cardiovascular Programming

The gut microbiota plays a critical yet distinct role in both CVD and cardiovascular programming, reflecting its influence across different life stages and disease trajectories. In the context of overt CVD, the gut microbiota plays a direct, pathogenic role. In CVD, microbial metabolites—such as TMAO, SCFAs, and tryptophan derivatives (e.g., indoxyl sulfate and indole-3-propionic acid)—exert direct effects by modulating inflammation, oxidative stress, endothelial function, and BP regulation [16,17,18].

**Figure 1:**
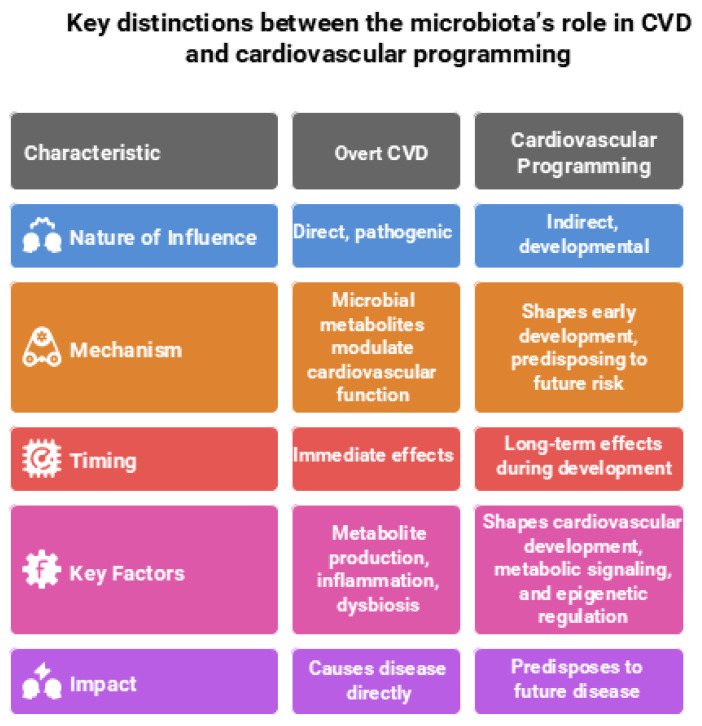
Comparison of the gut microbiota’s role in overt cardiovascular disease (CVD) and cardiovascular programming. Figure created using Napkin AI Image Generator [https://www.napkin.ai/ (accessed on 20 July 2025)].

In contrast, during cardiovascular programming—which encompasses long-term alterations in cardiovascular structure and function originating from early-life exposures—the gut microbiota plays a more indirect, developmental role. At this stage, the microbiota does not directly cause disease but instead shapes cardiovascular development, metabolic signaling, and epigenetic regulation during critical windows of growth. These early-life microbial influences establish physiological trajectories that predispose individuals to increased cardiovascular risk later in life [10,15]. Understanding these divergent roles is crucial for developing targeted interventions—whether for managing overt CVD or preventing cardiovascular disease through early-life microbiome modulation. Key distinctions between the microbiota’s role in CVD and cardiovascular programming are illustrated in Figure 1 and elaborated upon in the following sections.

### 3.1. The Role of Gut Microbiota in CVD

The gut microbiota contributes to atherosclerosis by promoting lipid accumulation, vascular inflammation, and immune activation while also driving endothelial dysfunction through impaired nitric oxide (NO) signaling, increased oxidative stress, and compromised vascular integrity [34]. These alterations create a pro-atherogenic environment that predisposes to CVD. Gut dysbiosis can worsen this process by increasing intestinal permeability, enabling bacterial components like lipopolysaccharides (LPS) to enter the bloodstream and provoke systemic inflammation and atherosclerotic plaque development [35,36]. Key microbial metabolites—including TMAO, SCFAs, and tryptophan derivatives—modulate vascular function, oxidative balance, and lipid and glucose metabolism. Collectively, these mechanisms contribute to endothelial injury, atherogenesis, thrombosis, and BP dysregulation [19,20].

#### 3.1.1. Gut Microbiota and Hypertension

Hypertension, a major modifiable risk factor for CVD, is closely linked to gut microbiota and derived metabolites [37,38]. A meta-analysis of 19 studies showed that hypertensive individuals tend to have lower microbial diversity, reduced levels of the SCFA-producing genus *Faecalibacterium*, and increased abundances of *Streptococcus* and *Enterococcus* [39]. Animal models, such as spontaneously hypertensive rats (SHRs), consistently exhibit gut dysbiosis and early intestinal pathologies—including reduced goblet cells, shortened villi, and loss of tight junction proteins—prior to the onset of hypertension [40]. Studies in which microbiota from hypertensive individuals were introduced into germ-free or normotensive animals show a pronounced rise in blood pressure, highlighting the causal role of gut microbial imbalance in hypertension [41]. Conversely, modifying gut microbiota through dietary interventions—such as the Mediterranean diet—and supplementation with probiotics, prebiotics, postbiotics, or FMT has been shown to lower BP and improve cardiovascular health in both humans and animal models [42,43,44,45]. These findings underscore a mechanistic link between gut microbiota and hypertension.

#### 3.1.2. TMAO and CVD

TMAO, a small amine oxide produced by gut microbial metabolism of dietary choline and L-carnitine, has been closely linked to atherosclerosis, thrombosis, and adverse cardiovascular outcomes [46]. In humans, elevated plasma TMAO levels are associated with an increased risk of myocardial infarction, heart failure, stroke, and all-cause mortality and have been proposed as a predictive biomarker for cardiovascular risk [47]. Mechanistically, TMAO promotes foam cell formation and cholesterol accumulation in macrophages, impairs endothelial function by increasing oxidative stress and reducing NO bioavailability, enhances platelet hyperreactivity, and triggers vascular inflammation [48,49,50]. In ApoE−/− and LDLr−/− mouse models, dietary supplementation with TMAO or its precursors accelerates atherosclerotic plaque development [51], while in hypertensive and atherosclerotic models, TMAO elevates reactive oxygen species (ROS) and disrupts endothelial integrity [52,53]. In chronic kidney disease (CKD) rat models, TMAO also contributes to endothelial dysfunction, inflammation, and elevated BP [54]. Notably, inhibition of trimethylamine (TMA), the microbial precursor of TMAO, has been shown to prevent these adverse effects, highlighting the TMA/TMAO metabolic pathway as a potential therapeutic target for CVD [54].

#### 3.1.3. Impact of Short-Chain Fatty Acids on CVD

Acetate, propionate, and butyrate, the main SCFAs, arise from microbial fermentation of fibers present in fruits, legumes, vegetables, and whole grains [55]. Key SCFA-producing bacteria include members of the *Ruminococcaceae* and *Lachnospiraceae* families, such as *Faecalibacterium prausnitzii*, *Akkermansia muciniphila*, etc., which are commonly associated with a healthy gut microbiome [56]. In both humans and animal models, SCFAs exert cardioprotective effects by regulating BP, reducing systemic inflammation, and improving endothelial function [55]. These actions are mediated in part through activation of SCFA-sensing G-protein-coupled receptors—GPR41, GPR43, and GPR109A—expressed in vascular, immune, and renal tissues [57,58]. Butyrate enhances gut barrier integrity and has anti-inflammatory properties, while propionate and acetate influence sympathetic nervous system activity, renin–angiotensin signaling, and vascular tone [59]. Reduced SCFA production—due to gut dysbiosis or low fiber intake—is linked to hypertension, atherosclerosis, and endothelial dysfunction [59]. Experimental studies demonstrate that enhancing SCFA availability through high-fiber diets or SCFA supplementation can lower BP and reduce cardiovascular risk [60], underscoring their therapeutic potential in CVD prevention.

#### 3.1.4. Inflammation and Immune Dysregulation

Inflammation and immune dysregulation are key pathways linking gut microbiota-derived metabolites to CVD, particularly in the context of CKD. Uremic toxins such as TMAO and several tryptophan metabolites—including indoxyl sulfate (IS), indoleacetic acid, and indoxyl-D-glucuronide—exert proinflammatory, prooxidant, and prothrombotic effects that contribute to CVD pathogenesis [61,62]. IS, for example, activates monocytes, enhances inflammatory cytokine release, promotes oxidative stress, and disrupts hemostasis, all of which drive vascular injury and atherosclerosis [63]. These microbial metabolites also interact with the aryl hydrocarbon receptor (AhR), a ligand-activated transcription factor involved in immune modulation [64]. Activation of AhR by tryptophan-derived catabolites promotes differentiation of pro-inflammatory helper T (Th)17 cells and suppresses regulatory T cells (Tregs), leading to a Th17/Treg imbalance implicated in hypertension and cardiovascular complications in CKD [65,66]. Additionally, IS and AhR signaling have been associated with thrombosis, a key mechanism in plaque rupture and acute CVD events. Other uremic toxins, such as p-cresyl sulfate, further exacerbate vascular inflammation by upregulating proinflammatory cytokines and adhesion molecules, thereby promoting atherogenesis [67]. Collectively, these findings highlight the central role of inflammation and immune imbalance in mediating the cardiovascular effects of gut-derived uremic toxins.

#### 3.1.5. Bile Acid Metabolism

Bile acid metabolism, shaped by both host physiology and gut microbiota activity, has gained attention for its role in CVD through its influence on lipid regulation, immune responses, and vascular homeostasis [68]. Evidence from both human and animal studies indicates that disruptions in bile acid composition—particularly elevated microbial-derived secondary bile acids—are linked to atherosclerosis, abnormal lipid profiles, and elevated BP. Bile acids exert systemic effects through receptors such as farnesoid X receptor (FXR) and TGR5, which regulate cholesterol transport, glucose metabolism, and inflammatory signaling pathways [69]. Gut dysbiosis can alter bile acid signaling, contributing to cardiometabolic dysfunction and vascular injury [68]. On the other hand, pharmacologic activation of FXR has been shown to lower plasma triglyceride levels and attenuate vascular inflammation in animal models [70], while TGR5 stimulation enhances endothelial function and reduces BP [71]. These findings highlight the importance of the gut microbiota–bile acid axis in CVD pathogenesis and its potential as a target for therapeutic intervention.

### 3.2. Impact of Early-Life Gut Microbiota on Cardiovascular Programming

Cardiovascular programming refers to the long-term alterations in cardiovascular structure and function that originate during early life, often triggered by adverse early-life exposures [10,72]. Maternal factors—including nutritional imbalance, toxins, stress, or illness—can disrupt the gut microbiota composition of both the mother and her offspring [21,73]. These early microbial disturbances exert indirect effects by modulating developmental pathways that govern cardiovascular maturation, thereby increasing the risk of hypertension, endothelial dysfunction, and CVD in later life [10].

Increasing data indicate that disturbances in the gut microbiota during early life may shape cardiovascular development indirectly by modulating oxidative stress [12], inflammatory and immune pathways [74], epigenetic modifications [15], and the renin–angiotensin–aldosterone system (RAAS) [75]. Microbial metabolites—such as SCFAs, TMAO, and secondary bile acids—do not act as immediate effectors but rather shape the developmental microenvironment. For instance, SCFAs can modulate gene expression via histone deacetylase (HDAC) inhibition, regulate immune maturation, and influence host receptor signaling. These microbiota-derived cues may gradually establish a pro-inflammatory and pro-hypertensive milieu, indirectly predisposing the offspring to cardiovascular dysfunction over time.

#### 3.2.1. Early-Life Gut Microbiota

Although microbial colonization of the neonatal gut begins immediately after birth, the gut microbiota continues to mature and diversify until it reaches an adult-like composition around 2–3 years of age [76]. The establishment of this early-life microbiome is shaped by a variety of maternal and perinatal factors, including gestational age, mode of delivery, maternal health status, feeding practices, antibiotic exposure, and broader ecological influences [77].

During pregnancy and lactation, maternal gut microbiota can influence the offspring’s microbial composition through vertical transmission and breast milk components, underscoring the pivotal role of maternal factors in shaping the early-life microbiome [73]. Notably, several conditions associated with the developmental origins of CVD—such as maternal obesity [78], diabetes [79], preterm birth [80], low birth weight [81], and maternal malnutrition [82]—are linked to alterations in early gut microbiota composition.

The early-life microbiome does not directly cause CVD but rather contributes indirectly by programming key physiological systems during critical developmental windows. These include immune system maturation and metabolic regulation—both of which are foundational to long-term cardiovascular health [83,84]. Disruptions in microbial development during this sensitive period may promote inflammation, oxidative stress, endothelial dysfunction, aberrant activation of the RAAS, and impaired metabolic signaling—features that gradually increase susceptibility to CVD later in life.

Additionally, prenatal exposure to environmental toxicants such as heavy metals, dioxins, and polycyclic aromatic hydrocarbons not only perturbs cardiovascular development but also alters the gut microbiota in ways that may further predispose the individual to hypertension and atherosclerosis in adulthood [85,86].

Collectively, these findings underscore that adverse maternal and environmental exposures shape the early-life gut microbiota, which in turn exerts indirect, developmental influences on the long-term risk of CVD through the programming of immune, metabolic, and vascular systems.

#### 3.2.2. Human and Animal Evidence of Cardiovascular Programming

Both epidemiological and experimental studies support the concept that adverse early-life exposures contribute to cardiovascular programming and elevate long-term risk for CVD. Human data have consistently linked early-life events—such as famine, maternal illness, pregnancy complications, medication use, environmental pollutants, and suboptimal nutrition—with increased risk of hypertension, obesity, dyslipidemia, type 2 diabetes, and CVD in adulthood [10,79,87,88,89]. For instance, offspring of diabetic pregnancies or those prenatally exposed to glucocorticoids or NSAIDs show higher susceptibility to cardiometabolic disorders [90,91]. Other early-life risk factors include low birth weight, gestational hypertension, short breastfeeding duration, rapid postnatal weight gain, vitamin D deficiency, and exposure to endocrine-disrupting chemicals [10,92,93].

While human studies establish strong associations, animal models provide critical causal insights into the timing, mechanisms, and potential for reprogramming. A growing body of animal research demonstrates that various maternal and perinatal exposures can induce cardiovascular programming, frequently accompanied by alterations in gut microbiota. These exposures include maternal high-fat [94] or high-fructose diets [95], uremia [96], antibiotic treatment [97], TMAO or other metabolite exposure [98], hypertension [99], Western-style diets [100], androgen excess [101], and dyslipidemia [102].

Animal studies indicate that gut microbial dysregulation can promote hypertension, vascular dysfunction, cardiac hypertrophy, and metabolic abnormalities such as obesity, insulin resistance, and fatty liver. Although such dysbiosis is implicated in a range of CVD—including coronary artery disease, cardiomyopathy, and heart failure [103]—its role in developmental programming of these conditions is not yet fully understood.

#### 3.2.3. Mechanisms Underlying Cardiovascular Programming

The fact that diverse early-life insults can result in similar cardiovascular outcomes—such as hypertension—in adulthood suggests the involvement of converging mechanistic pathways in the developmental origins of CVD. Several interrelated mechanisms have been implicated, including oxidative stress, NO deficiency, aberrant activation of the RAAS, chronic inflammation, gut microbiota dysbiosis, and epigenetic modifications [6,10,104,105]. Among these, gut microbiota dysbiosis appears to function as a central mediator, interacting with and modulating other key pathways.

Recent findings underscore the mechanistic contributions of the gut microbiota to cardiovascular programming. Microbial-derived metabolites such as SCFAs, TMAO, and uremic toxins can influence vascular function, immune regulation, and metabolic homeostasis [10]. SCFAs—particularly acetate, propionate, and butyrate—regulate BP via activation of specific receptors (e.g., GPR41, GPR43, and Olfr78) [56], affecting vasodilation and renin secretion [106]. Reduced SCFA levels during early life—often resulting from maternal high-fat diets or antibiotic exposure—have been linked to programmed hypertension [97], while SCFA supplementation during pregnancy and lactation has demonstrated protective effects [107]. TMAO has been shown in animal models to induce hypertension when administered during gestation, whereas inhibition of its formation prevents this outcome [95]. Likewise, in maternal CKD models, offspring hypertension was mitigated by maternal tryptophan supplementation, implicating modulation of the AhR signaling pathway [108]. Moreover, gut dysbiosis influences the RAAS, a critical regulator of cardiovascular homeostasis. Gut microbiota have been shown to affect ACE2 expression and intestinal amino acid transport, indicating a bidirectional relationship between microbial composition and RAAS function [109]. Therapeutic interventions targeting either gut microbiota (e.g., probiotics) or the RAAS have demonstrated reprogramming potential in animal models of developmental hypertension.

Taken together, these findings support a multifactorial model in which gut microbiota act not only as a mediator but also as an integrator of key molecular pathways—indirectly shaping cardiovascular development and increasing disease susceptibility later in life.

#### 3.2.4. Oxidative Stress and Its Link to Gut Microbiota in Cardiovascular Programming

Oxidative stress is a key mechanism underlying the developmental origins of hypertension and CVD. During pregnancy, suboptimal intrauterine conditions can lead to excessive production of reactive oxygen species (ROS), overwhelming the fetal antioxidant defense system and impairing cardiovascular development [110]. Various prenatal insults—including maternal illness, malnutrition, and environmental toxins—have been shown to induce oxidative stress and program hypertension in offspring [111]. This process involves increased activity of ROS-generating enzymes [112,113], reduced antioxidant capacity [114], impaired NO signaling [115], lipid peroxidation [116], DNA damage [117], and peroxynitrite formation [118].

Emerging evidence suggests that oxidative stress and gut microbiota dysbiosis are closely interconnected in cardiovascular programming. Animal models of maternal CKD [43], high-fructose [95], and high-fat diets [110] reveal that increased oxidative stress frequently coexists with altered gut microbiota in offspring predisposed to CVD. The gut microbiota contributes to redox homeostasis by modulating ROS signaling and maintaining gut–vascular integrity; disruption of this balance can amplify inflammation, compromise the intestinal barrier, and perpetuate microbial imbalance—a vicious cycle that enhances cardiovascular risk [119].

In a maternal CKD model, for instance, offspring hypertension was associated with both oxidative stress and gut dysbiosis, along with impaired NO signaling [117]. Perinatal treatment with resveratrol—an antioxidant with prebiotic properties—conferred protection by reducing oxidative stress and reshaping gut microbial composition, supporting a dual mechanism of reprogramming [96].

Together, these findings underscore oxidative stress and gut dysbiosis as interdependent and modifiable contributors to cardiovascular programming. This mechanistic insight supports the rationale for antioxidant-based interventions during pregnancy and lactation as a promising strategy to restore oxidative balance, reshape the gut microbiota, and prevent CVD of developmental origins.

## 4. Antioxidants as a Reprogramming Strategy

Antioxidants—whether dietary or synthetic—have emerged as promising reprogramming agents that target the intricate interplay between gut microbiota, oxidative stress, and cardiovascular programming. When administered during critical developmental windows, antioxidants can neutralize ROS, restore redox homeostasis, and modulate key signaling pathways that influence both host physiology and the gut microbial ecosystem [30,31,120,121]. Preclinical studies indicate that antioxidant-driven reprogramming occurs not only through ROS scavenging and enhancement of endogenous antioxidant defenses but also via modulation of gut microbiota. Antioxidants have been shown to shift microbial composition toward health-promoting profiles—reducing the production of harmful metabolites such as TMAO and indoxyl sulfate, while enhancing beneficial metabolites SCFAs. Through these integrated actions, antioxidants may interrupt the feed-forward loop linking gut dysbiosis, oxidative stress, and early-life programming of cardiovascular disease (CVD) [122].

While antioxidant interventions in adult CVD populations have produced inconsistent results—likely due to late-stage treatment, suboptimal dosing, or interindividual variability [23,24]—evidence from DOHaD models points to a more effective application: early-life antioxidant therapy as a reprogramming strategy. Targeting oxidative stress during fetal or early postnatal development may reset disease trajectories by preserving vascular integrity, shaping a balanced gut microbiome, and preventing maladaptive cardiovascular remodeling.

Dietary antioxidants are defined as naturally occurring food compounds that exhibit measurable antioxidant effects in vivo [123]. These encompass water-soluble molecules (e.g., vitamin C, glutathione, and lipoic acid) and lipid-soluble compounds (e.g., vitamins A and E, carotenoids, polyphenols, and coenzyme Q), many of which can additionally modulate gut microbial communities and their metabolites [30,31].

In addition to dietary sources, synthetic antioxidants such as N-acetylcysteine (NAC) and mitochondria-targeted agents like MitoQ have demonstrated efficacy in animal models of DOHaD [111,124]. Similarly, specific amino acids (e.g., L-arginine and L-citrulline) and hormones (e.g., melatonin) exhibit both antioxidant and microbiota-modulating properties, making them attractive dual-action reprogramming agents when used during pregnancy or early life [125,126].

The following section explores selected antioxidants investigated in the context of gut microbiota–cardiovascular interactions, highlighting their mechanisms of action and potential for translation into early-life therapeutic strategies to prevent CVD of developmental origin.

### 4.1. Vitamins and Micronutrients

Vitamins C and E, along with selenium and other micronutrients, possess antioxidant properties and have demonstrated beneficial effects on cardiovascular health [127]. Among these, vitamins C and E are the most extensively studied. Vitamin C, a water-soluble antioxidant, acts as a free radical scavenger and maintains redox balance [128], while vitamin E, a lipid-soluble antioxidant, inhibits oxidative enzymes and limits ROS generation [129]. Prenatal supplementation with either vitamin C or E has been shown to prevent hypertension in offspring subjected to maternal inflammatory stress, such as lipopolysaccharide (LPS) exposure [130,131]. Moreover, combined supplementation with vitamins C and E, selenium, and folic acid has conferred protection against hypertension and cardiovascular dysfunction in offspring from nutrient-restricted dams [132]. In a rabbit model, prenatal vitamin E administration attenuated maternal hypercholesterolemia-induced atherosclerosis in offspring [133].

Folic acid—a key B vitamin involved in one-carbon metabolism and DNA methylation—has demonstrated reprogramming effects by lowering hypertension and cardiovascular risk in offspring of protein-restricted pregnancies [134]. Similarly, maternal choline or betaine supplementation has mitigated cardiometabolic risks in offspring exposed to prenatal insults [135,136]. While these compounds act as methyl donors for epigenetic regulation [137,138], their role in preventing CKM syndrome via targeted epigenetic modulation remains incompletely understood.

Although these vitamins show protective effects on cardiovascular programming and are known to influence gut microbiota composition [30], their capacity to directly reprogram early-life microbiota and modulate gut–vascular signaling in developmental contexts has received limited investigation. Future studies are needed to clarify how micronutrient antioxidants might shape early microbiome development and contribute to long-term cardiovascular health.

### 4.2. Amino Acids

Perinatal amino acid supplementation has been explored in both clinical and experimental studies to improve pregnancy outcomes and fetal growth [139]. Several amino acids—such as arginine, citrulline, taurine, cysteine, glycine, tryptophan, and branched-chain amino acids (BCAAs)—possess antioxidant properties and play roles in cardiovascular and metabolic programming [140]. Arginine and citrulline are key precursors for NO production [141,142]. Arginine administration during lactation improved hepatic insulin signaling and suppressed gluconeogenesis in offspring [143]. Citrulline supplementation during pregnancy or lactation prevented offspring hypertension in models of antenatal dexamethasone exposure, maternal diabetes, and maternal CKD [144,145,146]. Its protective effect may involve modulation of hypertension-associated gut microbes such as Monoglobus and Streptococcus [147].

Taurine, a sulfur-containing amino acid, has shown consistent protective effects against hypertension and CVD programmed by various maternal insults [148,149,150,151]. In maternal CKD, perinatal taurine treatment reshaped the offspring gut microbiota—restoring *Bifidobacterium* and increasing *Asteroleplasma* and *Dehalobacterium*, while reducing *Erisipelatoclostridium*—with *Bifidobacterium* restoration contributing to its antihypertensive effect [151]. Cysteine, another sulfur-containing amino acid, protected offspring from hypertension by boosting hydrogen sulfide (H_2_S) production and promoting beneficial microbes (*Oscillibacter* and *Butyricicoccus*) while suppressing indole-producing bacteria (*Akkermansia* and *Alistipes*) and their metabolites [152].

Other amino acids, including glycine [153], BCAAs [154], and tryptophan [155], have also demonstrated reprogramming potential. In maternal CKD, tryptophan supplementation altered tryptophan-metabolizing microbes and modulated AhR signaling, contributing to protection against programmed hypertension [155]. Given the critical role of maternal–fetal amino acid metabolism in development, it is essential to clarify the specific functions and interactions of individual amino acids in cardiovascular programming and microbiota modulation to ensure safe and effective reprogramming strategies.

### 4.3. Melatonin

As a key hormone in pregnancy, melatonin (N-acetyl-5-methoxytryptamine) supports fetal development [156]. Beyond its physiological roles, it functions as a potent antioxidant, with both melatonin and its metabolites reducing oxidative stress by scavenging reactive oxygen species, promoting antioxidant enzyme expression, and increasing nitric oxide levels [157,158]. It also interacts bidirectionally with the gut microbiota—modulating microbial composition, alleviating dysbiosis, and serving as a signaling mediator between gut health and host circadian and physiological regulation [159]. Given these properties, perinatal melatonin supplementation has emerged as a promising reprogramming strategy to prevent adult-onset diseases linked to DOHaD [160].

The beneficial effects of maternal melatonin therapy have been supported be different models against cardiovascular programming-induced offspring hypertension [161,162,163] and CVD [164]. It is noteworthy that melatonin’s cardioprotective attributes are mainly ascribed to its various antioxidant actions, including the inhibition of mitochondrial respiratory chain complex, the activation of nuclear factor erythroid 2-related factor 2 (Nrf2), and the suppression of inflammatory cytokine release [165,166,167,168].

Previous studies show that melatonin modulates early-life gut microbiota and metabolism, enhancing resistance to inflammation. Maternal melatonin supplementation increased Firmicutes abundance, elevated gut melatonin and butyrate levels, and reduced intestinal inflammation in neonatal mice [169]. In young CKD rats, melatonin therapy restored microbial α-diversity, increased Proteobacteria and Roseburia abundance, and reversed CKD-related TMAO dysregulation, linking its protective effects to gut microbiota modulation [170].

However, clinical use of melatonin during pregnancy remains uncertain [171,172], despite its use in some neonatal conditions [173]. Further translational research is urgently needed to clarify its long-term effects on cardiovascular programming.

### 4.4. Polyphenols

Polyphenols, a diverse group of phytochemicals abundant in plant-based foods, are recognized for their antioxidant capacity and emerging cardiovascular benefits [174,175]. These compounds counteract oxidative stress via multiple pathways, such as scavenging reactive oxygen species, enhancing nitric oxide bioavailability through nitric oxide synthase activation, chelating pro-oxidant metal ions, and stimulating endogenous antioxidant defenses [176,177]. Beyond redox regulation, polyphenols also interact with the gut microbiota, acting as microbiota-modulating agents that promote a healthier microbial balance [178,179].

Although numerous meta-analyses have linked polyphenol-rich diets to reduced CVD risk [180,181,182,183], only a fraction of individual polyphenols has been examined in developmental models of cardiovascular programming. Polyphenols are broadly categorized into flavonoids and non-flavonoids [184]. Among the flavonoids, quercetin and epigallocatechin gallate (EGCG) have shown promise in mitigating hypertension in adult offspring exposed to prenatal insults, such as maternal high-fat intake [185] or antenatal glucocorticoid exposure [186].

Resveratrol, a well-studied non-flavonoid polyphenol, is notable for its dual antioxidant and prebiotic functions [187]. Resveratrol exerts cardioprotective effects primarily through mechanisms including antioxidant activity, anti-inflammatory signaling, activation of MAPK1 pathway, and modulation of endothelial function [188]. Evidence from experimental models suggests that early-life resveratrol exposure confers long-term protection against hypertension and cardiovascular dysfunction [189,190,191]. In maternal models of CKD, high-fructose diets, and metabolic overload, perinatal resveratrol supplementation improved gut microbial composition in offspring—marked by elevated *Bifidobacterium* and *Lactobacillus* levels, increased microbial diversity, and normalization of the *Firmicutes*-to-*Bacteroidetes* ratio [96,192,193]. These microbial shifts are linked to reduced risk of hypertension and suggest that resveratrol may act as a microbiota-targeted intervention to counter CKD programming.

Despite their therapeutic potential, clinical application of polyphenols is hindered by poor bioavailability and inter-individual differences in absorption and metabolism [194]. This complexity underscores the need for further investigation into the specific roles of individual polyphenols and their gut microbiota interactions in cardiovascular programming. Optimizing delivery strategies and identifying responsive phenotypes may enhance translational outcomes in future preventive medicine.

### 4.5. SCFAs

Produced primarily by gut microbes, SCFAs influence oxidative stress and support fetal growth [195,196]. Perinatal administration of acetate, butyrate, or propionate has been found to mitigate programmed hypertension in offspring subjected to maternal high-fructose intake [96], minocycline [197], or tryptophan-deficient [198] conditions. In the high-fructose model, acetate lowered plasma TMA levels, reduced the TMA/TMAO ratio, and upregulated renal SCFA receptors [196]. In the minocycline model, acetate reduced hypertension and increased probiotic genera such as *Roseburia* and *Bifidobacterium* [197]. Butyrate was protective in the tryptophan-deficient model, associated with gut microbiota modulation, enhanced SCFA receptor expression, and restoration of RAS balance [198]. A comparative study in high-fructose-fed dams found that while both butyrate and propionate raised circulating SCFA levels, butyrate more strongly impacted TMAO metabolism and NO signaling, whereas propionate primarily influenced gut microbiota composition [199]. These findings suggest SCFAs function not only as antioxidants but also as key microbiota-mediated modulators in hypertension reprogramming.

### 4.6. N-Acetylcysteine

NAC, a naturally occurring antioxidant in *Allium* plants, serves as a precursor to glutathione [200] and a stable L-cysteine analogue involved in hydrogen sulfide (H_2_S) synthesis [201]. Perinatal NAC therapy has been shown to prevent offspring hypertension in various early-life insult models [202,203,204,205]. Reprogramming effects of NAC have been linked to reduced lipid peroxidation, increased glutathione levels, decreased renal oxidative stress, and enhanced NO bioavailability [202,203,204,205]. In spontaneously hypertensive rats (SHRs), maternal NAC treatment was linked to increased abundance of *Bifidobacterium* and its parent phylum *Actinobacteria* [202], known sulfur-oxidizing bacteria (SOB) [206]. As NAC increased both *Actinobacteria* and fecal thiosulfate—an H_2_S oxidation product—its protective effects may involve enhanced SOB activity and thiosulfate production. Additionally, in a maternal suramin exposure model, NAC administered during gestation and lactation reprogrammed hypertension and reduced renal oxidative stress [204]. These findings highlight NAC’s dual function as an antioxidant and microbiota modulator in developmental hypertension prevention.

### 4.7. Synthetic Antioxidants

In addition to natural antioxidants, a variety of synthetic antioxidants have been investigated in animal models for their potential to reprogram cardiovascular risks, particularly hypertension of developmental origins. Among these, lipid peroxidation inhibitors such as lazaroids have shown promising results—maternal lazaroid therapy effectively prevented elevated BP in adult offspring exposed to maternal protein restriction [207].

Another notable class is NRF2 (nuclear factor erythroid 2-related factor 2) activators, such as dimethyl fumarate (DMF). NRF2 is a redox-sensitive transcription factor that, upon release from its inhibitor KEAP1, translocates to the nucleus and activates the expression of antioxidant genes via the antioxidant response element (ARE) [208,209]. In a rat model involving maternal dexamethasone exposure and postnatal high-fat diet, DMF treatment prevented the development of offspring hypertension [210], highlighting NRF2 activators as potential therapeutic agents against oxidative-stress-driven cardiovascular programming.

Although synthetic antioxidants like tempol and MitoQ have been extensively studied in oxidative-stress-related models [211,212], their efficacy in reprogramming cardiovascular outcomes remains largely unexplored.

Beyond classical antioxidants, several pharmacological agents have demonstrated antioxidant-like effects by modulating the ROS–NO balance through regulation of asymmetric dimethylarginine (ADMA) metabolism. Agents such as rosuvastatin [213], metformin [214], salvianolic acid A [215], oxymatrine [216], telmisartan [217], and GLP-1 receptor agonists [218] can reduce ADMA levels either by inhibiting its synthesis or enhancing its degradation. Among these, metformin has been shown to prevent offspring hypertension in a maternal high-fructose and post-weaning high-fat diet model in parallel with its ADMA-lowering effect [219]. While metformin is also known to modulate gut microbiota [220], it remains unclear whether this contributes to its reprogramming effects.

Collectively, these findings underscore the therapeutic promise of synthetic antioxidants and related agents as reprogramming strategies to mitigate cardiovascular risks originating from early-life oxidative stress. However, further studies are warranted to clarify their mechanisms—particularly interactions with gut microbiota—and to confirm their long-term efficacy and safety in offspring.

### 4.8. Others

Recent evidence indicates that probiotics and prebiotics exhibit antioxidant and anti-inflammatory properties, helping to counteract oxidative stress [221,222]. Consequently, interventions aimed at modulating the gut microbiota—including the use of probiotics, prebiotics, postbiotics, and microbial-metabolite-targeted strategies—hold potential as approaches to prevent cardiovascular programming driven by oxidative stress, though further research is needed [10].

While animal models have supported the role of oxidative stress as a viable target for developmental reprogramming, translation into human studies remains limited. A key barrier is the ethical and logistical challenge of conducting interventional trials involving pregnant or lactating women. In this context, breast milk emerges as a practical and biologically potent reprogramming tool, owing to its rich antioxidant profile [223] and distinct microbiome, which plays a pivotal role in shaping the infant gut microbiota [224,225]. Universally acknowledged as the optimal source of infant nutrition, breast milk contains an array of nutrients and bioactive compounds—including hormones, growth factors, cytokines, microRNAs, metabolites, prebiotics, probiotics, and oligosaccharides—that collectively contribute to infant development and immune programming [226,227].

Importantly, breastfeeding has been associated with reduced long-term cardiovascular risk, particularly in preterm infants [228,229]. Given current global recommendations for exclusive breastfeeding during the first six months of life [230], its dual capacity to deliver antioxidants and beneficial microbes positions breast milk as a compelling, naturally integrated strategy for preventing early-life cardiovascular programming. A concise overview of the key findings is presented in Table 1.

## 5. Conclusions and Future Perspective

This narrative review highlights current evidence on the interplay between oxidative stress and gut microbiota during early life, emphasizing the potential of antioxidants as a reprogramming strategy to prevent cardiovascular programming and reduce the risk of CVD later in life. While antioxidant-based interventions are promising, most current studies are associative in nature and often constrained by small sample sizes, heterogeneous methodologies for microbiome profiling, and inadequate control for key confounders such as maternal diet, socioeconomic status, and antibiotic exposure. These limitations underscore the need for more targeted and translational research. Key knowledge gaps include identifying the optimal antioxidant mechanisms, timing, and formulations [120]; elucidating the bidirectional interactions between oxidative stress and gut microbiota within the DOHaD framework [10]; and bridging preclinical findings to clinical application [231].

Targeting oxidative stress in early life is an attractive reprogramming strategy, but several important limitations must be considered. Antioxidant therapies—whether oral or intravenous—circulate systemically and may reduce ROS levels below the physiological range in healthy tissues, potentially disrupting redox-sensitive signaling pathways essential for normal pregnancy and fetal development [232,233]. Moreover, the oxidant–antioxidant system is closely interconnected with inflammatory and immune networks, and perturbations in this balance could lead to immediate or long-term adverse outcomes for both the mother and fetus. Therefore, antioxidant supplementation during gestation or lactation should be approached cautiously and reserved for cases with clear evidence of oxidative imbalance, rather than used as routine prophylaxis.

Moreover, excessive antioxidant exposure may paradoxically induce “antioxidant stress”, disrupting redox homeostasis, and potentially contributing to adverse developmental outcomes [234]. While antioxidant interventions are biologically plausible as reprogramming agents, excessive or indiscriminate use—particularly at higher doses of vitamins and micronutrients—may disrupt physiological redox signaling, interfere with inflammatory and immune networks, and pose risks to both mother and fetus. Clinical trials have yielded inconsistent results, possibly due to suboptimal timing, limited bioavailability, or failure to target oxidative pathways most relevant to developmental programming. The optimal dose, timing, and therapeutic window for antioxidant-based reprogramming remain undefined, and comprehensive clinical and experimental studies elucidating the mechanisms linking early-life antioxidant exposure to adult cardiovascular outcomes are lacking. Future research should identify precise developmental windows (e.g., prenatal vs. pre-weaning), delineate organ-specific redox-sensitive pathways involved in cardiovascular programming, and match antioxidant strategies to the right dose and timing for safe and effective outcomes. These efforts should be coupled with longitudinal birth cohort studies integrated with mechanistic experimentation and multi-omics approaches to disentangle the interplay between microbiota-derived metabolites, redox balance, and cardiovascular phenotypes.

In parallel, significant gaps exist in our understanding of how oxidative stress and gut microbiota interact within the DOHaD paradigm. Although both are known contributors to early-life CVD risk, their dynamic interplay is seldom investigated as an integrated system. The mechanisms by which oxidative stress influences microbial composition and, conversely, how microbiota-derived metabolites modulate host redox balance are still poorly understood.

A longitudinal birth cohort should be established to profile newborn gut microbiota at birth using metagenomic sequencing, with participants followed into adulthood through periodic cardiovascular assessments. This design would enable identification of early-life microbial signatures predictive of later CVD, integrating environmental, dietary, genetic, and maternal factors—including oxidative status and microbiota composition during pregnancy—to generate critical evidence linking perinatal microbiota to long-term cardiovascular risk and to pinpoint modifiable early-life targets for prevention.

Therapeutic strategies that concurrently target oxidative stress and gut dysbiosis—such as probiotics, prebiotics, synbiotics, and postbiotics—offer promising yet underexplored avenues. Addressing these research gaps will pave the way for the development of precision-based, integrative interventions aimed at preventing CVD from its origins in early life.

**Figure 2:**
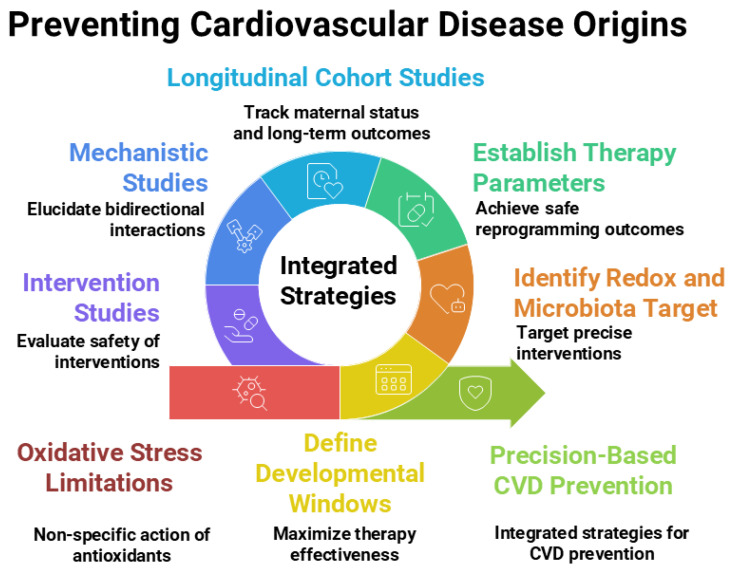
Integrated strategies, current research gaps, and future directions related to antioxidants and gut microbiota within the Developmental Origins of Health and Disease (DOHaD) framework for preventing the developmental origins of cardiovascular disease. Figure created using Napkin AI Image Generator [https://www.napkin.ai/ (accessed on 20 July 2025)].

Moving forward, a multidisciplinary approach will be essential—engaging experts from developmental biology, free radical medicine, cardiovascular physiology, nutrition, and microbiology—to unravel these complex interconnections and translate them into safe, effective strategies for lifelong cardiovascular health. Figure 2 illustrates the current research gaps and outlines future research directions.

## Figures and Tables

**Table 1:** Summary of antioxidant-based reprogramming strategies targeting gut microbiota, oxidative stress, and cardiovascular programming.

Category	Examples	Main Mechanisms	Microbiota Modulation	Key Evidence	Translational Considerations
Vitamins and Micronutrients	Vitamin C, Vitamin E, Selenium, Folic Acid, Choline, Betaine	ROS scavenging, lipid peroxidation inhibition, methyl donor for epigenetic regulation	Limited direct reprogramming studies; known to influence microbiota composition	Prevented hypertension and CVD in offspring under maternal inflammation, nutrient restriction, hypercholesterolemia	Widely available; human data limited for developmental microbiota–vascular effects
Amino Acids	Arginine, Citrulline, Taurine, Cysteine, Glycine, BCAAs, Tryptophan	NO production, H_2_S production, antioxidant defense enhancement	Alters hypertension-associated microbes; restores beneficial genera (*Bifidobacterium*, *Oscillibacter*); reduces harmful bacteria (*Akkermansia*, *Alistipes*)	Prevented hypertension in maternal CKD, diabetes, dexamethasone exposure; modulated AhR signaling	Requires understanding of dose/timing; maternal–fetal amino acid metabolism critical
Melatonin	Endogenous hormone	ROS scavenging, ↑ antioxidant enzymes, ↑ NO, Nrf2 activation, ↓ inflammation	Restores microbial diversity; ↑ *Firmicutes*, *Roseburia*; ↓ TMAO dysregulation	Prevented hypertension/CVD in multiple DOHaD models; improved gut and vascular health	Safe in some neonatal uses; pregnancy safety uncertain; further long-term data needed
Polyphenols	Quercetin, EGCG, Resveratrol	ROS scavenging, NO bioavailability ↑, metal chelation, endogenous antioxidant activation	↑ *Bifidobacterium*, *Lactobacillus*, diversity; normalize *Firmicutes/Bacteroidetes* ratio	Resveratrol protected against hypertension in CKD, high-fructose, metabolic overload models	Poor bioavailability; interindividual absorption variability; targeted delivery strategies needed
SCFAs	Acetate, Butyrate, Propionate	Antioxidant effects, SCFA receptor activation, RAS balance restoration, TMAO metabolism modulation	↑ *Roseburia*, *Bifidobacterium*; enhance microbial diversity	Prevented hypertension in high-fructose, minocycline, tryptophan-deficient models	Potential as microbiota–metabolite therapy; diet-dependent production
N-acetylcysteine (NAC)	NAC (from Allium plants)	Glutathione precursor, H_2_S synthesis, ROS reduction, ↑ NO bioavailability	↑ *Actinobacteria*, *Bifidobacterium*, sulfur-oxidizing bacteria; ↑ fecal thiosulfate	Prevented hypertension in maternal suramin, SHR, CKD models	Dual antioxidant–microbiota actions; promising for oxidative-stress-driven hypertension
Synthetic Antioxidants	Lazaroids, DMF (Nrf2 activator), Tempol, MitoQ	Lipid peroxidation inhibition, antioxidant gene activation, ADMA metabolism modulation	Limited data; some agents (metformin) modulate microbiota	DMF prevented hypertension in dexamethasone + high-fat model; metformin reduced ADMA and hypertension	Many agents already clinically available; need microbiota mechanism studies
Others	Probiotics, Prebiotics, Postbiotics, Breast Milk	Antioxidant and anti-inflammatory effects, microbiome shaping	Direct microbiota delivery and modulation	Breastfeeding linked to lower long-term CVD risk; probiotics/prebiotics show benefit in animal models	Ethical barriers in pregnancy trials; breast milk offers natural antioxidant + probiotic synergy

ROS—Reactive Oxygen Species; CVD—Cardiovascular Disease; NO—Nitric Oxide; H_2_S—Hydrogen Sulfide; BCAAs—Branched-Chain Amino Acids; AhR—Aryl Hydrocarbon Receptor; DOHaD—Developmental Origins of Health and Disease; TMAO—Trimethylamine N-oxide; SCFAs—Short-Chain Fatty Acids; NAC—N-acetylcysteine; SHR—Spontaneously Hypertensive Rat; DMF—Dimethyl Fumarate; ADMA—Asymmetric Dimethylarginine; EGCG—Epigallocatechin Gallate; RAAS—Renin–Angiotensin–Aldosterone System.

## Data Availability

Data are contained within the article.

## Abbreviations

The following abbreviations are used in this manuscript:

ROS	Reactive Oxygen Species
BP	Blood Pressure
CVD	Cardiovascular Disease
CKD	Chronic Kidney Disease
NO	Nitric Oxide
H_2_S	Hydrogen Sulfide
BCAAs	Branched-Chain Amino Acids
AhR	Aryl Hydrocarbon Receptor
DOHaD	Developmental Origins of Health and Disease
TMA	Trimethylamine
TMAO	Trimethylamine N-oxide
SCFAs	Short-Chain Fatty Acids
NAC	N-acetylcysteine
SHR	Spontaneously Hypertensive Rat
DMF	Dimethyl Fumarate
ADMA	Asymmetric Dimethylarginine
EGCG	Epigallocatechin Gallate
RAAS	Renin–Angiotensin–Aldosterone System
LPS	Lipopolysaccharide
NRF2	Nuclear factor erythroid 2-related factor 2
FXR	Farnesoid X Receptor
TGR5	G protein–coupled Bile Acid Receptor 1
GPR41	G-protein-coupled Receptor 41
GPR43	G-protein-coupled Receptor 43
GPR109A	G-protein-coupled Receptor 109A
